# Databases for lncRNAs: a comparative evaluation of emerging tools

**DOI:** 10.1261/rna.044040.113

**Published:** 2014-11

**Authors:** Sabrina Fritah, Simone P. Niclou, Francisco Azuaje

**Affiliations:** NorLux Neuro-Oncology Laboratory, Department of Oncology, Centre de Recherche Public de la Santé (CRP-Santé), Luxembourg L-1526, Luxembourg

**Keywords:** noncoding RNAs, lncRNAs, databases

## Abstract

The vast majority of the human transcriptome does not code for proteins. Advances in transcriptome arrays and deep sequencing are giving rise to a fast accumulation of large data sets, particularly of long noncoding RNAs (lncRNAs). Although it is clear that individual lncRNAs may play important and diverse biological roles, there is a large gap between the number of existing lncRNAs and their known relation to molecular/cellular function. This and related information have recently been gathered in several databases dedicated to lncRNA research. Here, we review the content of general and more specialized databases on lncRNAs. We evaluate these resources in terms of the quality of annotations, the reporting of validated or predicted molecular associations, and their integration with other resources and computational analysis tools. We illustrate our findings using known and novel cancer-related lncRNAs. Finally, we discuss limitations and highlight potential future directions for these databases to help delineating functions associated with lncRNAs.

## INTRODUCTION

The genomes of humans and other mammalian organisms encode a wide variety of noncoding RNAs (ncRNAs), which have been implicated in diverse mechanisms regulating biological function. Among the range of RNA molecules, long noncoding RNAs (lncRNAs) are increasingly being associated with networks of epigenetic and post-transcriptional control in health and disease. LncRNAs constitute a diverse class of transcripts that are larger than 200 nucleotides and do not serve as templates for proteins. Their size can vary from hundreds of base pairs to tens of kilobases. They are often transcribed by polymerase II, and, like messenger RNAs, can be post-transcriptionally modified by capping, polyadenylation, and splicing (Guttman et al. 2009; Beaulieu et al. 2012; Guttman and Rinn 2012; Yin et al. 2012).

Although until recently the prevailing view was that lncRNA transcription was a rare event, now it is estimated that 70% of our genome is transcribed, while only 1.2% represents protein-coding sequences (Human Genome Sequencing Consortium International 2004; Djebali et al. 2012). These estimates are possible thanks to advances in transcriptomics and next-generation sequencing. In contrast to small ncRNAs, lncRNAs are less evolutionarily conserved at the sequence level and have been divided into five biotypes in relation to their proximity to protein-coding genes: sense, antisense, bidirectional, intronic, and intergenic (Ponting et al. 2009; Gibb et al. 2011). Further categorization of lncRNAs relies on their molecular features. LncRNAs can function as signaling molecules and reflect promoter activity or can regulate chromatin structure (Brown et al. 1992; Andersen and Panning 2003; Foulds et al. 2010). They can work as molecular guides and/or scaffolds for RNP (ribonucleoprotein) complexes. Finally, by sequestering regulatory RNAs or proteins, lncRNAs can act as decoys (Tripathi et al. 2010; Leucci et al. 2013).

**FIGURE 1.**
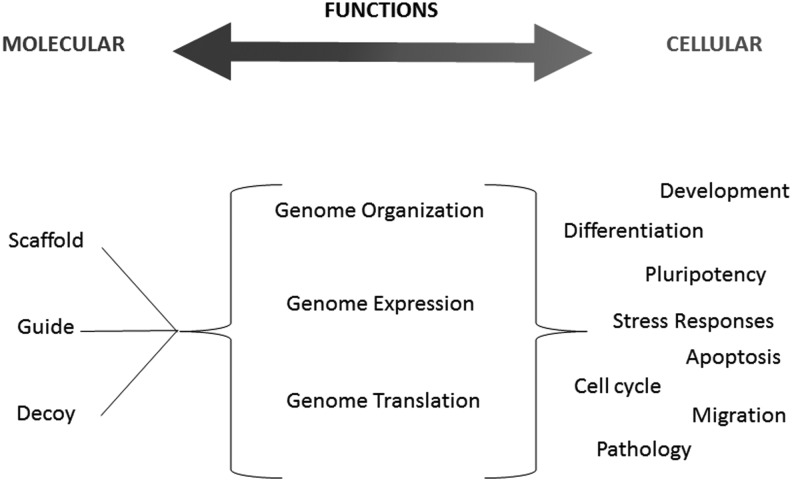
Overview of lncRNAs functions.

At the cellular level, one of the best characterized roles of lncRNAs is epigenetic regulation; for a recent review, see Mercer and Mattick (2013). LncRNAs can bind a large number of chromatin modifying proteins and guide them to remodel the structure and/or expression of their neighboring genes (*cis*). However, these chromatin-associated lncRNAs may also act in *trans*, as exemplified by Xist. The latter binds to many long-distance locations in the X chromosome, thereby inducing its entire silencing for dosage compensation (Brown et al. 1991; Brockdorff et al. 1992; Herzing et al. 1997). Besides epigenetic control, lncRNAs can regulate transcription, alternative splicing, RNA translation, and organize important structures for RNA processing such as nuclear speckles (Tripathi et al. 2010, 2012; Zong et al. 2011; Yoon et al. 2013). Figure 1 summarizes the different molecular and cellular functions of lncRNAs.

LncRNAs have been implicated in cell identity as their expression is more cell-type specific or tissue specific than that of protein isoforms (Cabili et al. 2011). Importantly, lncRNA isoforms may exercise different roles depending on their subcellular location. Indeed, a nuclear isoform of PTEN antisense transcript and its cytosolic counterparts have opposite effects on PTEN expression, due to differential sequestration of small ncRNAs within the two cellular compartments (Poliseno et al. 2010; Jalali et al. 2012). Owing to these diverse molecular mechanisms, lncRNAs are now known as important regulators of fundamental processes such as development, imprinting, and cell differentiation (Kretz 2013; Lee and Bartolomei 2013; Lv et al. 2013). They are also involved in stress responses to heat, hypoxia, or DNA damage (Jolly et al. 2004; Bertozzi et al. 2011; Wu et al. 2012; Place and Noonan 2013; Wan et al. 2013; Yang et al. 2013a; Zhang et al. 2013).

Research on the relationship between lncRNAs and pathophysiology, mainly on genetic disorders and oncology, is growing fast. Genome-wide association studies have revealed that most single-nucleotide polymorphisms are located in nonprotein-coding regions that encompass lncRNA genes (Cariaso and Lennon 2012; Cheetham et al. 2013). Interestingly, some lncRNAs are transcribed from disease risk loci, and recently it was shown that a polymorphism affecting a lncRNA predisposes to thyroid carcinoma (Jendrzejewski et al. 2012).

Several studies have deciphered an involvement of lncRNAs in key tumorigenesis steps: Some lncRNAs act as tumor suppressors, others participate in cellular replicative immortality, or even regulate angiogenesis and metastasis. More recently, Xist has been proposed as a possible therapeutic molecule in Down syndrome and hematologic cancer (Jiang et al. 2013; Yildirim et al. 2013).

Despite these advances, the regulatory roles of only a few lncRNAs have been biologically characterized to date. On another level, we are confronted with a fast accumulation of large-scale data sets and novel computing tools, which will eventually enable the generation of new hypotheses about the roles of lncRNAs in different disease phenotypes. LncRNA information resources are needed to address unmet knowledge discovery needs of the research community.

Multiple high-quality resources of annotations are needed to identify and characterize lncRNAs in genomic studies. As the molecular function of lncRNAs is mediated through interactions with other RNA species and proteins, it is important to have access to large-scale data sets that report or computationally predict such relevant associations, in particular with regard to disease-related processes. In this context, existing genomics data could be reannotated in terms of noncoding genes or transcripts to begin to understand their putative clinical relevance. In this respect, a recent analysis of the Cancer Genome Atlas (TCGA) data identified potentially clinically relevant noncoding transcripts. The expression of specific lncRNAs appears to be linked to patient survival, copy number alteration, or histological subgrouping in glioblastoma as well as in lung, ovarian, and prostate cancers. This analysis also provided clues about the potential role of lncRNAs as prostate cancer drivers (Du et al. 2013).

To further exploit existing and emerging data sets, it is essential that the scientific community is aware of the scope, advantages, and limitations of available lncRNA data resources. To facilitate this effort, here we review relevant databases that compile and integrate different types of lncRNA-related information. Moreover, we provide recommendations on their application based on a critical assessment of their content coverage and quality, as well as of their predictive potential. We divide this evaluation into fundamental qualitative aspects, which range from the detection and annotation of lncRNAs and their association with other RNAs, to the computational tools that these databases offer to enable lncRNA research. We illustrate the usage, challenges, and potential of the databases with an application case in the oncology area. Finally, we discuss key advantages and limitations of the databases investigated, and provide an outlook for the future exploitation of lncRNA-oriented databases.

## DETECTION AND ANNOTATION OF lncRNAs

Transcription of lncRNAs was first evidenced with traditional cloning methods without any further detection of translation products. A major progress in experimental detection of noncoding gene expression came with microarrays and tilling arrays (targeted versus contiguous sets of sequences, respectively), and more recently, with deep sequencing approaches (Okazaki et al. 2002; Carninci et al. 2005; Kapranov et al. 2005).

The discovery of lncRNA function through their interaction with other molecular species is based on novel experimental techniques which rely on the isolation of a component of interest (protein, RNA) and the identification of interacting partners (RNA, protein, and/or DNA). The identification step is often performed with high-throughput sequencing and/or mass spectrometry. RIP (RNA immunoprecipitation) and HITS/PAR-CLIP (HIgh-Throughput Sequencing of RNA/PhotoActivatable-Ribonucleoside-CrossLinking and ImmunoPrecipitation) technologies allow the identification of multiple RNAs linked to a protein. Conversely, using RNA pull-down or ChIRP/CHART techniques, proteins and DNA sequences associated with a particular lncRNA can be identified. When using bioinformatics tools, it is important to distinguish the limitations of these techniques, especially in terms of prediction of putative indirect or direct (binding) relationships.

In the computational identification of lncRNAs, a traditional premise has been that the sequences of candidate lncRNAs exhibit limited protein-coding potential. Thus, for example, those sequences that show open reading frames (ORFs) smaller than a predefined number of amino acids, e.g., 30 amino acids, were proposed as potential lncRNAs (Okazaki et al. 2002; Kapranov et al. 2005; Katayama et al. 2005). The reliability of such predictions can be enhanced by estimating the level of conservation of these ORFs across species. Limited conservation between species is seen as additional evidence of the noncoding potential of the investigated sequences. Additional genomic features can be integrated to further refine the list of potential lncRNAs. For example, a recent study showed that the majority of lncRNA genes tend to be located within 10 kb from protein-coding genes (Jia et al. 2010).

Several bioinformatics approaches to lncRNA identification based on the reannotation of gene expression array probes have been proposed. Typically, such a reannotation process involves the mapping of microarray probe sets to databases, such as Ensembl, which provide annotations on the noncoding potential of the probes. The resulting candidate lncRNAs can be functionally characterized by estimating different types of biological associations with known protein-coding genes. One such representative approach consists of applying “guilt-by-association” algorithms in the context of lncRNA–gene association networks. For example, based on expression profiles, correlations between lncRNAs and protein-coding genes have been analyzed in diverse experimental conditions to assign putative functions from characterized coding genes to candidate lncRNAs (Liao et al. 2011; Guo et al. 2013).

Associations between lncRNAs, other regulatory RNAs and proteins can be computationally inferred with existing approaches to predict targets for transcription factors (TFs) and microRNAs (miRNAs). These techniques are usually based on the identification of functional similarity patterns extracted from sequences (DNA or RNA motifs), of gene coexpression, and of evolutionary conservation relationships (Kel et al. 2003; Muniategui et al. 2013). The computational prediction of interactions can also involve machine learning models built on training data sets that contain relatively large collections of known lncRNA–RNA interactions, together with instances defined as noninteracting pairs. The models are trained to classify RNA–RNA or RNA–protein associations according to specific biological features and interaction “labeling” criteria. For example, Glazko et al. (2012) generated computational models that distinguish between lncRNAs binding and not binding the polycomb repressive complex 2 (PRC2). In this model, lncRNAs and PRC2 proteins were represented by different sequence and structural features found to be statistically associated with lncRNA–PRC2 interactions.

## OVERVIEW OF AVAILABLE lncRNA DATABASES

**FIGURE 2.**
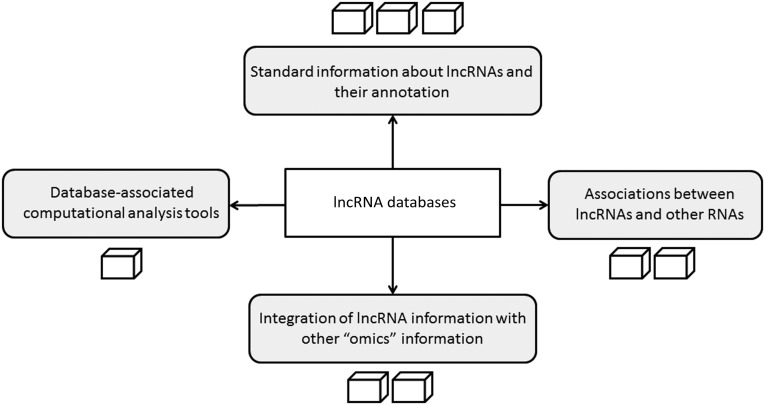
Key lncRNA database aspects evaluated. The number of cubes associated with each aspect reflects relative amounts of content available in the databases investigated in this article.

Although lncRNAs are becoming increasingly available in public data sets, literature-supported evidence of their biological activity is still relatively limited. Recently, diverse resources dedicated to lncRNAs have been developed, which differ in data coverage and quality. Therefore, we evaluated lncRNA databases that met the following criteria: (a) The database has been published in peer-review journals and (b) the database is available through a web-based searchable interface (Table 1). Our main objective was to assess these resources according to key fundamental informational aspects relevant to data content and integrative capability (Fig. 2). The resulting comparative characteristics will inform readers in their future choices based on research-specific needs or requirements. It was not our intention to identify the “best” databases or perform an exhaustive comparison of their data content. A software-oriented evaluation, an analysis of primary data quality or a user-driven evaluation of interface functionality are also outside the scope of this review. All the database-specific information reported here were available via their websites as of 25 July 2013.

**TABLE 1.**
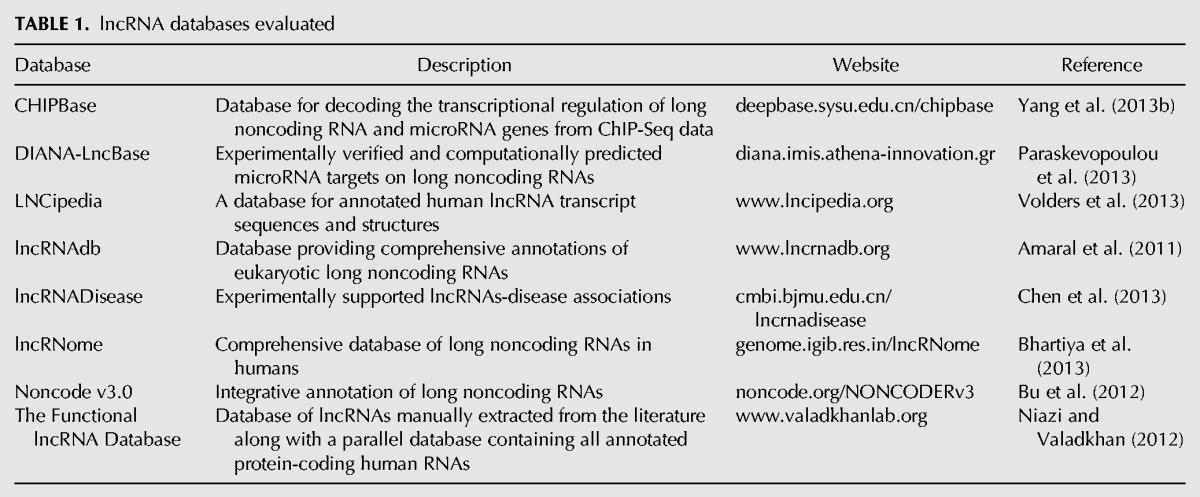
lncRNA databases evaluated

### Fundamental database information and lncRNA annotations

The number of lncRNAs stored in the databases varies from <2000 to >70,000 transcripts. For instance, the largest database (Noncode v3.0) stores >73,000 transcripts. Not all databases provided sufficient information about the total number and origin source of the transcripts. DIANA-LncBase contains the largest number of experimentally verified lncRNAs (2958 transcripts), and is the largest repository of putative (computationally) predicted lncRNAs (>56,000 transcripts). The majority of the databases, except CHIPBase and the Functional lncRNA Database, allow users to download all or part of their data as files. This is useful to facilitate further specialized analyses or the development of new computing tools. All the databases automatically generate visualizations of query results as lists or tables. In addition, most of them offer alternative graphical visualizations, such as diagrams or plots (CHIPBase, DIANA-LncBase, LNCipedia, Noncode v3.0, and lncRNome).

The stored lncRNAs and their biological annotations are obtained from the literature, computational predictions, or primary data repositories. A key example of the latter is the GENCODE project (Derrien et al. 2012), part of the ENCODE project (The ENCODE Project Consortium 2012), which offers accurate annotations of the human genome, including noncoding transcripts. Conversely, the Functional lncRNA Database and lncRNADisease entirely rely on manually curated, literature-extracted annotations. DIANA-LncBase is the only database that specifies the incorporation of lncRNA annotations originating from the literature, from computational predictions, and from primary data repositories. All the databases include lncRNA annotations that are supported by experimental evidence, i.e., we did not find database that solely rely on computational evidence.

All the databases provide lncRNAs identified in humans. Some of them also include information specific to mouse (CHIPBase, DIANA-lncBase, lncRNAdb, Noncode v3.0, and the Functional lncRNA Database), as well as other model organisms (CHIPBase, DIANA-lncBase, lncRNAdb, and the Functional lncRNA Database). In particular, LncRNAdb and Noncode v3.0 databases cover lncRNAs expressed in a large number of other species, from yeast to plants. The following databases offer information on the cell or tissue specificity of lncRNAs: CHIPBase, DIANA-LncBase, lncRNAdb, Noncode v3.0, and lncRNome. Only lncRNAdb and Noncode v3.0 designate the cellular localization of the lncRNAs.

Different databases describe the lncRNAs in terms of biological functional annotations, including validated and putative functional associations: DIANA-lncBase, lncRNAdb, Noncode v3.0, and lncRNome. The Functional lncRNA Database stores annotations exclusively based on validated functional lncRNAs. Most of the databases, DIANA-lncBase, lncRNAdb, lncRNADisease, Noncode v3.0, and lncRNome, provide information about putative or validated associations between lncRNAs and diseases. These annotations are extracted from the literature or other databases.

### Linking lncRNAs to other molecules

New advances in fundamental and translational research will require an accurate understanding of the functional connection between lncRNAs and other RNAs, including both protein-coding and noncoding RNAs (Table 2). In our set of investigated databases, only CHIPBase and lncRNome describe associations between lncRNAs and coding RNAs. Such relationships are mainly based on the identification of the nearest coding genes to the lncRNAs. The spectrum of databases that specify experimental evidence about lncRNA–transcription factors (TF) associations is wider: CHIPBase, lncRNAdb, lncRNADisease, and lncRNome.

**TABLE 2.**
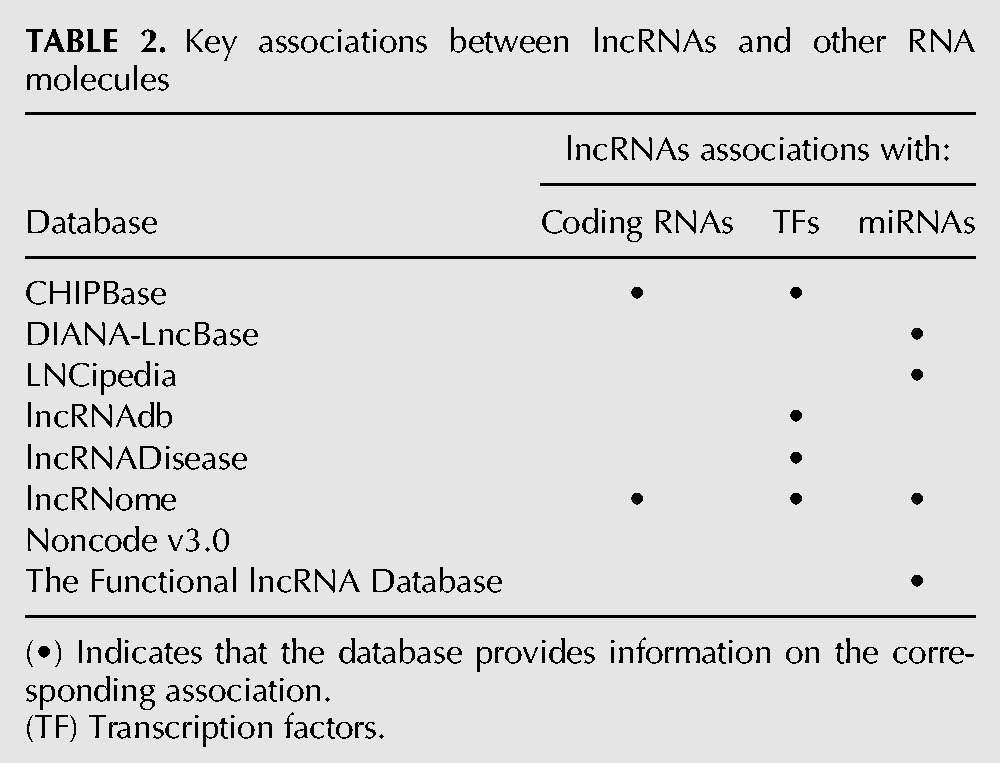
Key associations between lncRNAs and other RNA molecules

Information about associations between lncRNAs and other noncoding RNAs is only available in some of the databases evaluated and is based on different sources of experimental evidence. The following databases specify lncRNA–miRNA associations: DIANA-lncBase, LNCipedia, the Functional lncRNA Database, and lncRNome. DIANA-lncBase and lncRNome offer the most diverse set of sources of experimental evidence to define such associations, including HITS-CLIP and PAR-CLIP data. The Functional lncRNA Database describes lncRNAs that contain potential miRNA precursors. None of the resources examined provide associations between lncRNAs and other types of noncoding RNAs (outside miRNAs). Noncode v3.0, however, describes computational matches between lncRNAs and similar transcript sequences.

### Integration of lncRNA databases and other ‘omics’ data sets

A useful requirement of lncRNA databases for enabling fundamental and translational research is their integration with additional biological information, which can be inferred computationally from lncRNA-specific data, stored in third-party repositories or mined from the literature.

Different types of “omics” data are relevant to assist in the characterization of lncRNAs. For example, information on the protein-coding potential of candidate lncRNAs is typically predicted through the application of bioinformatics techniques. This involves the estimation of “coding potential scores,” such as those proposed by Kong et al. (2007) and Bu et al. (2012), and which are based on the analysis of sequence-derived features of the transcripts. Among the resources examined here, LNCipedia, Noncode v3.0, the Functional lncRNA Database, and lncRNome offer indicators of the protein-coding potential of the lncRNAs stored in these databases. In addition to sequence-based calculations, LNCipedia integrates mass spectrometry data to measure the coding potential of lncRNAs.

As part of the characterization of putative lncRNAs, researchers can benefit from additional information about the reported genomic categorization of the candidate transcripts. On the basis of the genomic position that the transcripts occupy, lncRNAs are usually assigned to two main categories: genic and intergenic transcripts. The former can be further categorized into exonic, intronic, and overlapping candidate lncRNAs. DIANA-lncBase, lncRNAdb, Noncode v3.0, and lncRNome offer such categorizations as part of their lncRNA annotations.

The spectrum of “omic” information that lncRNA databases can provide also ranges from genomic and gene expression to epigenetics to structural information. All the databases evaluated, with the exception of DIANA-lncBase, display sequence-level information of their lncRNAs. In addition, all databases describe the genomic location of the lncRNAs, i.e., their genomic coordinates. Snapshots of or direct links to published gene expression data are included in CHIPBase, DIANA-lncBase, lncRNAdb, lncRNADisease, Noncode v3.0, and lncRNome.

To study the regulation of lncRNAs, as well as their potential regulatory roles, databases will increasingly provide information on epigenetic activity, such as that derived from ChIP-Seq experiments. Currently, CHIPBase and lncRNome are the only databases sharing this type of data through their websites. CHIPBase comprises 543 ChIP-Seq peak data sets for 252 different transcription factors, whereas lncRNome encompasses 11,790 histone modifications and methylation data in lncRNA promoters. Another aspect that will require further attention is the inclusion of information about the secondary structure of the lncRNAs. LNCipedia and lncRNome already describe lncRNAs in terms of computationally predicted RNA structures and motifs. As these molecules rarely code for proteins, it has been hypothesized that they are less conserved at the sequence level than mRNAs, which renders phylogenic studies of lncRNAs more challenging. Interspecies conservation of secondary structure may be more informative to investigate the functional importance of lncRNAs (Johnsson et al. 2014).

As the size and diversity of data sets increase, lncRNA databases will require stronger couplings with third-party information resources. This includes literature databases, other specialized databases, genome browsers, and computing analysis platforms. For instance, all the assessed databases establish links between their lncRNA entries and the literature as supporting evidence for their annotations. Most of these databases also directly interface with other external resources, such as genomic and phenotype-related databases hosted at the NCBI (National Center for Biotechnology Information) (ncbi 2013).

### Database-associated computational analysis tools

Another important requirement in the development of lncRNA databases is the integration of lncRNA information with diverse computational tools to allow further characterization of the lncRNAs, as well as the prediction of novel biological associations. Such tools can be either directly deployed on the database website or externally linked to it through different software integration techniques.

Although the main emphasis of the databases evaluated here is the storage and search of lncRNA information together with basic visualization functionality, many of them already offer computational techniques to support data analysis. This comprises the automated identification of candidate lncRNAs (lncRNADisease), the computational estimation of putative functional associations between lncRNA and other types of RNA (DIANA-lncBase), and the statistical detection of sets of lncRNAs that are highly implicated in specific biological processes or pathways (CHIPBase).

The fast growing nature of lncRNA research will demand open, community-driven approaches to storing and sharing information. This includes, for example, the dynamic incorporation of emerging evidence on experimentally validated lncRNAs and functional characterizations. LncRNAdb and lncRNADisease currently allow researchers to submit new lncRNAs and associated information, which subsequently undergo some level of human expert verification and integration into the databases. All the databases examined (except the Functional lncRNA Database) provide a dedicated section with user-oriented documentation, which describes the database content, website functionality, or usage guidelines.

**FIGURE 3.**
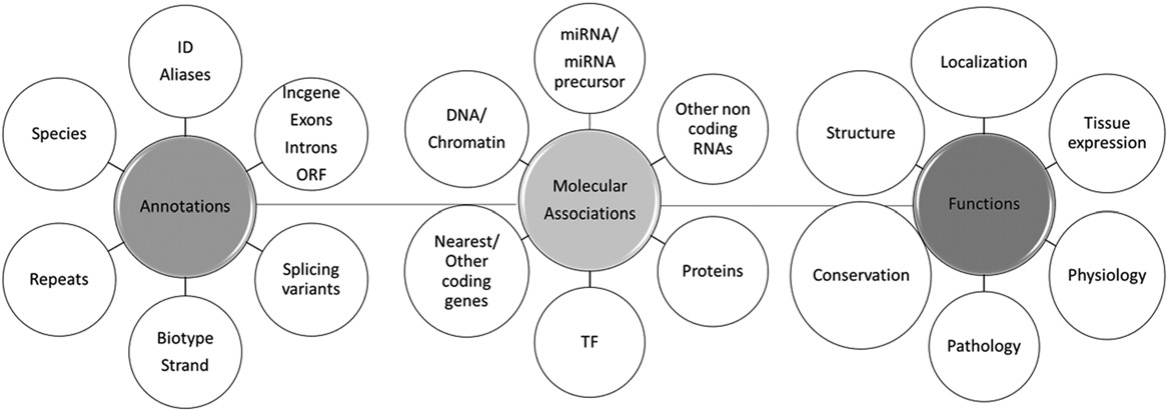
Organization of lncRNA database contents. LncRNAs display multiple annotations: ID corresponds to the lncRNA Identifier (Ensembl, Noncode, Refseq), Aliases (lncRNA name); lncRNAs can be defined as different biotypes (sense, antisense, bidirectional, intronic, and intergenic) and are transcribed from different DNA strands. (TF) transcription factors.

Figure 3 offers a global integrated view of the different database content dimensions examined here. This framework also guides the application study illustrated in the next section.

## APPLICATION CASE IN CANCER RESEARCH

In order to offer a more practical view of these resources and their application, we extracted (from each database) information relevant to two cancer-associated lncRNAs: a well-characterized lncRNA (Meg3) and a lncRNA of unknown function (transcript ENSG00000228288, in chromosome 1) that was identified in a prostate cancer data set.

### Application example using a known lncRNA

#### Annotations

**FIGURE 4.**
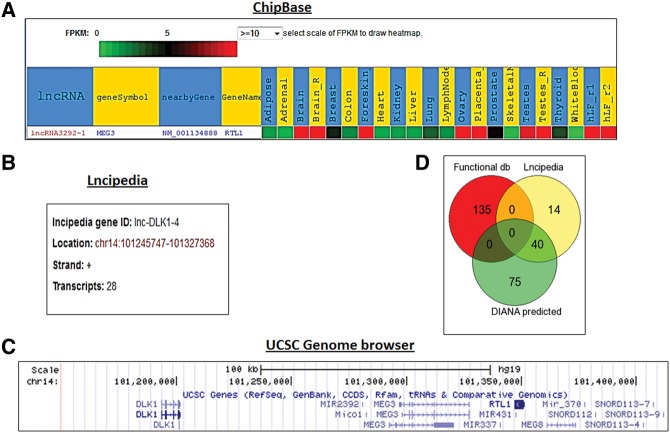
Example of discrepancies across databases using the lncRNA Meg3 as query. *Meg3* is related to the protein-coding gene *Rtl1* in ChIPBase (*A*), whereas it is associated with *Dlk1* in LNCipedia (*B*). (*C*) Representation of the MEG3 containing genomic region with the UCSC Genome browser. (*D*) Venn diagram showing overlap of microRNAs associated with Meg3 in LNCipedia, DIANA-LncBase, and the Functional lncRNA Database.

In the case of the well-characterized lncRNA, Meg3, we compared database-extracted information in terms of consistency and complementarity. Meg3 entry was found in all databases, with similar genomic locations, positive strand transcription, and as a long intergenic biotype (lincRNA). However, we observed less consistency in more detailed annotations. Gene aliases differ among ChIPBase (*Rtl1*), LNCipedia (*Dlk1*), and lncRNAdb (*Gtl2*) (Fig. 4A,B). In order to understand this discrepancy, we visualized the *Meg3* genomic region in the UCSC Genome Browser (Kent et al. 2002) and localized the nearest coding genes (Fig. 4C). A possible explanation for this difference in nearest gene definition may be due to taking (or not) into account the strand of transcription (Fig. 4C). In terms of transcript variants, *Meg3* corresponds to 28 isoforms in lncRNome and LNCipedia databases, whereas in Noncode and ChIPBase this transcript is associated with 41 variants. Interestingly, the Functional lncRNA Database gives information about repeat elements contained in *Meg3*, which may be helpful to design specific probes.

#### Molecular associations

Apart from indicating the nearest protein-coding gene, the databases report or predict molecular associations between a given lncRNA and DNA, RNA, or proteins. ChIPBase offers the largest number of TF–lncRNA associations (15 in total). Other databases (lncRNAdb and lncRNADisease) could link *Meg3* to two proteins, although no common substrate was found in the outputs. As lncRNAs interact with or give rise to miRNAs, these associations are also listed in four databases. The Functional lncRNA Database, DIANA-LncBase, and LNCipedia report 135, 115, and 54 miRNA–Meg3 associations, respectively. Although the Functional lncRNA Database contains the highest number of miRNAs linked to Meg3, no overlap was found with the other two databases (Fig. 4D). DIANA-LncBase and LNCipedia display similarities in the evidence sources used to establish the associations. A possible explanation for the observed differences is that most of these molecular associations are based on computational predictions. Lastly, the lncRNome database links Meg3 to seven small RNA clusters (without detailed information about their identities).

#### Function

To assess the roles of Meg3 in pathophysiology, we first queried lncRNADisease and the Functional lncRNA Database, which are specialized in disease- and function-related content. As indicated in these databases, previous research has shown that Meg3 may function as a tumor suppressor in a number of cancers and acts through the regulation of p53 expression (Zhou et al. 2007, 2012). Other databases with more generic content also contain this information based on the literature. Two databases (Noncode and ChIPBase) show tissue expression of Meg3. lncRNome revealed a number of SNPs in *Meg3*. Moreover, information on subcellular localizations (lncRNAdb), conservation (LNCipedia and lncRNAdb), or protein-coding potential (LNCipedia, Noncode, lncRNome) are useful to decipher cellular function of *Meg3*. Lastly, prediction of three-dimensional structure of Meg3 (LNCipedia and lncRNome) could be helpful to define functional domains in the different Meg3 isoforms.

### Application example using a novel lncRNA

The novel lncRNA (ENSG00000228288) was found in three databases: lncRNAdb, lncRNome, and LNCipedia. In the latter, this lncRNA is only found using its alias, i.e., KDM5B-AS1. Surprisingly, although DIANA-LncBase and Noncode contain the highest number of lncRNAs, this novel antisense transcript was not present. The three databases containing this lncRNA report similar annotations, number of alternative transcripts (three in total), coding potential, and structural features. While LNCipedia and LncRNAdatabase do not indicate any associations with miRNAs, lncRNome refers to two small RNA clusters associated with KDM5B-AS1. Using lncRNome, we could identify chromatin modifications and SNPs associated with this lncRNA. Another unexpected finding was that lncRNAdb and lncRNome already include a literature link to this relatively novel lncRNA entry.

In summary, a plethora of information can be extracted from the lncRNA databases investigated here. The results retrieved are similar in terms of broad annotation information, mainly on genomic locations. Generic lncRNA databases (e.g., LNCipedia, lncRNome, and lncRNAdb) display complementarity in molecular association features. Table 3 recapitulates the general content and features of each database. For both relatively well-known and novel lncRNAs, diverse information could be obtained, which may be useful to extend the characterization of their potential functional mechanisms. Altogether, these case studies show that lncRNA resources are useful to support or even to drive experimental research.

**TABLE 3.**
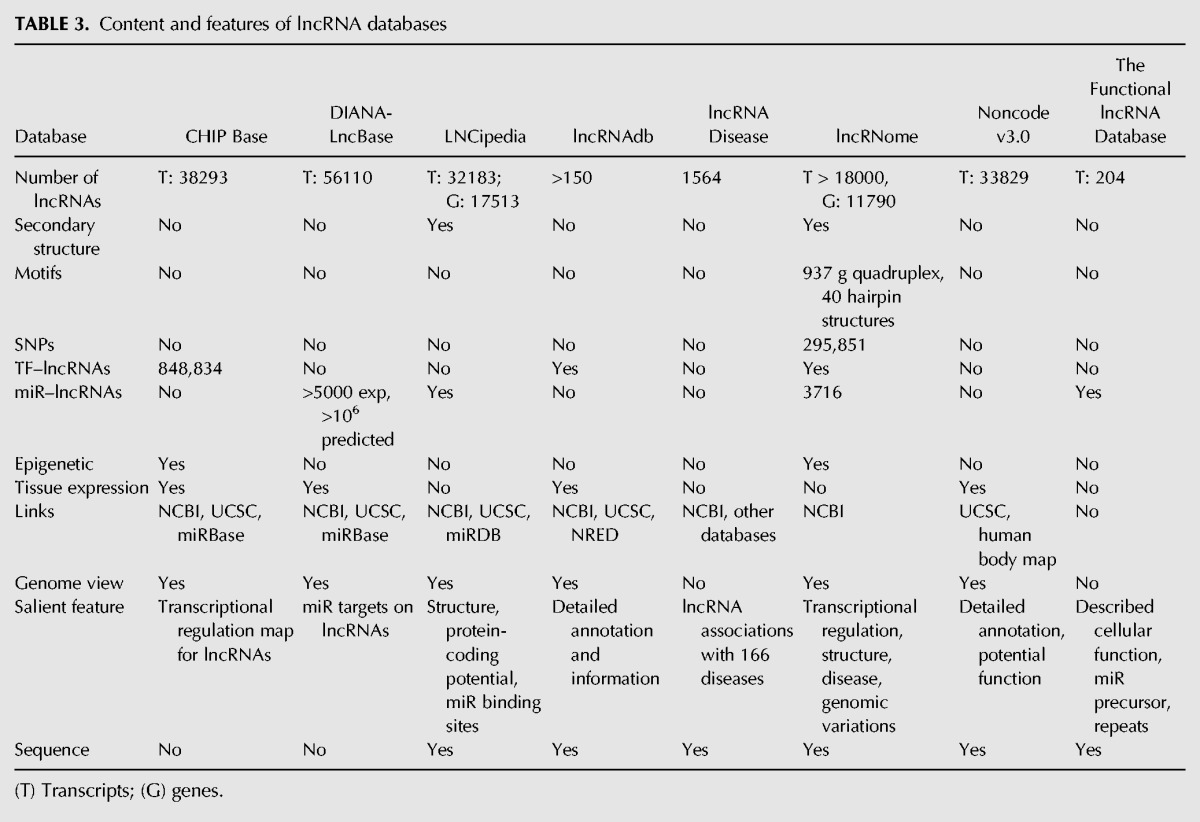
Content and features of lncRNA databases

## LIMITATIONS AND UNMET NEEDS

Although these resources offer considerable amounts of information for lncRNA research, they show various limitations that require careful attention and indicate potential future directions to improve these tools. We observed major discrepancies across databases regarding the detailed annotations of the lncRNAs. Indeed, lncRNA names are often related to their neighboring coding genes, which could be different between databases. When analyzing lncRNAs in terms of their coexpression relationship with coding genes, this parameter could influence the output. Timely updates in genome annotations and databases should help to solve this issue. Also it would be important to make a distinction between long noncoding RNA genes (lnc genes) and lncRNAs. Lnc genes correspond to transcriptional genomic units of lncRNAs. Lnc genes display exonic/intronic structures and produce splicing isoforms of lncRNAs. A clear distinction between gene ID and transcript ID for lncRNAs will improve the understanding of lncRNA biogenesis and splicing. This idea has been recently implemented in the latest version of the Noncode database (Xie et al. 2014).

While most databases allow searches with multiple entries including: Refseq, Noncode and Ensembl IDs, in lncRNAdb queries are restricted to lncRNA names, which may be problematic when analyzing new or putative lncRNAs.

Regarding information about molecular associations, we observed a poor overlap in the results from the different databases. This is probably explained by the different data sources or algorithms used to predict these interactions. As only exemplified by the DIANA-LncBase, it is important to distinguish experimentally based molecular associations from computationally predicted ones. Additionally, as lncRNAs often act as decoys, a comprehensive view of interactions between lncRNAs and other types of RNAs or proteins is still required. Although the databases mainly include links between lncRNAs and miRNAs, other types of noncoding RNAs, such as snoRNAs and circRNAs, are likely to become more relevant research topics. Ideally, it would be useful to further specify the nature of their associations as lncRNAs could interact with or give rise to small ncRNAs (miRNAs and snoRNAs). Moreover, except for ChIPBase and lncRNome, interactions between lncRNAs and chromatin are not included in the databases investigated. This information is important because lncRNAs may exert important functions in epigenetic regulation and chromatin dynamics. Therefore, ChIP-Seq data could be better exploited to describe novel lncRNA function.

We found other relatively minor limitations in the current content of lncRNA databases. Although the biogenesis of lncRNAs involves either polymerase II or polymerase III, this feature is not presently included (Dieci et al. 2007; Wu et al. 2012). Also, even when databases such as lncRNAdb and Noncode currently indicate subcellular location of lncRNAs extracted from literature, this type of annotation remains limited. One could take advantage of recent ribosome profiling data (Chew et al. 2013; Guttman et al. 2013) to further evaluate the proportion of lncRNAs that are exported into the cytoplasm. This could also improve the description of the bifunctional lncRNA biotype.

## CONCLUSIONS

Until recently, the expression of noncoding sequences was largely considered as transcriptional noise. The notion that lncRNAs may play important functions has now gained solid ground. It merits substantial research efforts to investigate their biological activity and potential functionality, which may lead to potential translational applications.

Advances in transcriptomics and high-throughput sequencing are facilitating the fast accumulation of lncRNA data sets, which are being collected and organized in diverse databases. In this fast growing field, lncRNA databases help to delineate transcript–function relationships. Thus, when using these resources, we recommend to start with general content databases, such as lncRNome and LNCipedia, which offer a good compromise between coverage and depth of annotations. In general, existing databases provide adequate links between lncRNAs and relevant literature sources. We found that this is specially the case of lncRNAdb, Noncode, and lncRNome. With regard to molecular associations, ChIPBase, DIANA-LncBase, LNCipedia, and lncRNome are complementary. Therefore, we suggest researchers to use several databases and compare overlaps between the molecular interactions retrieved.

Despite the importance of these resources, we also identified some limitations in their current content, particularly in connection with the extent and granularity of the annotations available, and with the accuracy of the molecular associations reported. In the future, we should expect that more precise annotations at the level of individual lncRNAs and their interaction networks will allow their further exploitation within integrative data mining platforms. This will in part mirror the development of miRNA research.

During the review of this manuscript, additional resources were released for lncRNA research. LncRNA Map, Starbase v2.0, and LncRNAtor give insights into the potential regulatory roles of human lncRNAs and their interaction with miRNAs, as well as sRNAs (LncRNA Map), and proteins (Starbase v2.0 and LncRNAtor). In addition, LncRNAtor provides information on coexpression between mRNAs and lncRNAs in various tissues (Chan et al. 2014; Li et al. 2014; Park et al. 2014). Moreover, an updated version of the Noncode database is now available as Noncode v4.0 (Xie et al. 2014). Also we note that, apart from the resources reviewed here, other specific tools and databases exist, such as PLncDB (plant related lncRNAs) (Jin et al. 2013), NRED (noncoding expression database) (Dinger et al. 2009), and Linc2go (Liu et al. 2013).

In conclusion, comprehensive views of the potential molecular and cellular functions of lncRNAs will provide new insights into genetic disorders and other multifactorial conditions. In this endeavor, a deeper integration of these databases with information about the potential biological relevance of lncRNAs will be essential.
